# mHealth Intervention Promoting Cardiovascular Health Among African-Americans: Recruitment and Baseline Characteristics of a Pilot Study

**DOI:** 10.2196/resprot.8842

**Published:** 2018-01-31

**Authors:** LaPrincess C Brewer, Sarah Jenkins, Kandace Lackore, Jacqueline Johnson, Clarence Jones, Lisa A Cooper, Carmen Radecki Breitkopf, Sharonne N Hayes, Christi Patten

**Affiliations:** ^1^ Department of Cardiovascular Medicine Mayo Clinic College of Medicine Rochester, MN United States; ^2^ Division of Biomedical Statistics and Informatics Department of Health Sciences Research Mayo Clinic Rochester, MN United States; ^3^ Christ's Church of the Jesus Hour Rochester, MN United States; ^4^ Southside Community Health Services, Incorporated Minneapolis, MN United States; ^5^ Division of General Internal Medicine Department of Medicine Johns Hopkins University School of Medicine Baltimore, MD United States; ^6^ Department of Health Sciences Research Mayo Clinic Rochester, MN United States; ^7^ Department of Psychiatry and Psychology Mayo Clinic College of Medicine Rochester, MN United States

**Keywords:** mHealth intervention, community-based participatory research, cardiovascular disease, cardiovascular health, health disparities, African-Americans, faith-based intervention

## Abstract

**Background:**

Mobile health (mHealth) interventions are promising avenues to promote cardiovascular (CV) health among African-Americans (AAs) and culturally tailored technology-based interventions are emerging for this population.

**Objective:**

The objectives of this study were to use a community-based participatory research (CBPR) approach to recruit AAs into a pilot intervention study of an innovative mHealth CV health promotion program and to characterize technology use patterns and eHealth literacy (EHL).

**Methods:**

Community partners from five predominately AA churches in southeast Minnesota collaborated with our academic institution to recruit AA congregants into the pilot study. Field notes as well as communications between the study team and community partners were used to design the recruitment strategy and its implementation with a goal of enrolling 50 participants. At its core, the recruitment strategy included community kickoff events to detail the state-of-the-art nature of the mHealth intervention components, the utility of CV health assessments (physical examination, laboratory studies and surveys) and the participants’ role in advancing our understanding of the efficacy of mHealth interventions among racial/ethnic minority groups. Detailed recruitment data were documented throughout the study. A self-administered, electronic survey measured sociodemographics, technology use and EHL (eHEALS scale).

**Results:**

A total of 50 participants (70% women) from five AA churches were recruited over a one-month period. The majority (>90%) of participants reported using some form of mobile technology with all utilizing these technologies within their homes. Greater than half (60% [30/50]) reported being “very comfortable” with mobile technologies. Overall, participants had high EHL (84.8% [39/46] with eHEALS score ≥26) with no differences by sex.

**Conclusions:**

This study illustrates the feasibility and success of a CBPR approach in recruiting AAs into mHealth intervention research and contributes to the growing body of evidence that AAs have high EHL, are high-users of mobile technologies, and thus are likely to be receptive to mHealth interventions.

## Introduction

African-American (AA) participation in mobile health (mHealth) studies is expanding in parallel to their increased adoption of mobile technologies [1-6]. Recent studies have integrated culturally tailored web-based platforms [7,8], monitoring technologies [9], and text-messaging [10-12] strategies to address key cardiovascular disease (CVD) risk factors among AAs, but few have attempted to translate existing effective evidence-based, community-based behavioral interventions into mHealth interventions within this population. Furthermore, there is a need to better understand the technology use patterns of AAs and their eHealth literacy (EHL) (perceived skills to effectively utilize and apply electronic health information) as these factors have been associated with healthy lifestyle behavioral change[13-15].

AA faith communities offer a promising avenue to foster recruitment into and deliver mHealth interventions as technology integration into health promotion activities at church may facilitate their implementation, dissemination and sustainability [9,16]. In addition, community-based recruitment approaches have cultivated trust between researchers and increased enrollment and retention rates in AAs [1,17,18]. We previously created a face-to-face, community-based, CVD prevention program among AA congregants in Minnesota (MN) which was successful in improving cardiovascular (CV) health knowledge and promoting CV health [19]. Post-intervention analyses revealed keen interest in translating the face-to-face program into an mHealth intervention to increase its accessibility and reach within AA faith communities [20]. Use of a community-based participatory research (CBPR) strategy bolstered our intervention recruitment and implementation efforts and fostered acceptability of the overall program by the AA community members[18,20]. Thus, we hypothesized that the ongoing use of a CBPR approach as a means to engage AAs in the translational design and development of an mHealth intervention, acting to springboard from a face-to-face program, would maximize recruitment within the AA community.

In this report, we describe our recruitment strategy design incorporating a CBPR approach in addition to its effectiveness and challenges. We also report participant baseline characteristics, technology use patterns, and EHL.

## Methods

### Study Design and Description

The Fostering African-American Improvement in Total Health (FAITH!) program is a behavioral theory–informed, culturally tailored, community-based CV health and wellness program implemented as an academic-community partnership with our institution and local AA churches [19,20]. As previously reported, the FAITH! face-to-face intervention targeted multiple CV risk factors (ie, hypertension, dyslipidemia, diabetes) among AA faith communities. Participants demonstrated improvements in CV health literacy and CV health metrics (blood pressure, body mass index [BMI]) along with positive trends between self-efficacy and health behaviors. Participant evaluations indicated an interest in integrating mobile technology or the Internet into the program to optimize dissemination and sustainability [20]. In response to the program’s demonstrated effectiveness and its overall participant acceptability, the study team and community partners mutually decided that design of an mHealth intervention would enhance the program’s reach to the AA community while creating a less resource-intensive yet effective tool for AA churches. Using a CBPR approach with community partners from five predominantly AA churches in the Rochester and Minneapolis-St Paul (MSP), MN areas, we jointly developed an innovative, interactive, on-demand lifestyle intervention in the form of an app through an iterative and formative research design process to ensure its usability, satisfaction and cultural relevance for the AA faith community. The purpose of the app as a whole was to deliver health education and motivational support to users to improve CV health. The app-based intervention (*FAITH! App*) included a 10-week core series of multimedia education modules addressing key CV risk factors as well as interactive self-quizzes, self-monitoring (diet and physical activity) and social networking (discussion sharing board). The intervention was intended for participants to follow a weekly schedule of each education module concentrating on each CV behavior or risk factor.

Within the current *FAITH! App* pilot study, health assessments were conducted at baseline and 6-months post-intervention at local community health clubs (in Rochester and MSP, MN) with the assistance of a trained nursing team. These assessments included collection of CV health risk factors: anthropometrics (BMI, waist circumference), blood pressure measurements (average of three sitting readings, by oscillometric automated blood pressure monitor, Bp TRU BPM-100) and laboratory studies (total cholesterol, glucose by fingerstick). Electronic surveys were administered at baseline, mid-intervention, post-intervention and 6-months post-intervention including further measures of CV health behaviors (measured by an adapted version of the National Cancer Institute fruit and vegetable all-day screener [22] and the International Physical Activity Questionnaire-short form [23]). Primary outcomes included self-efficacy (diet and physical activity [24,25]), CV health knowledge and CV health (risk factors and behaviors [19]). Additional survey items included self-reported mobile technology use, Internet access, and EHL [13-15]. EHL was evaluated using the eHealth Literacy Scale (eHEALS) which consists of eight items scored on a 5-point Likert scale which assesses an individual’s perception of their ability to understand and apply electronic health information [13]. The sum of all items ranges from 8 to 40 with higher scores reflecting a higher level of EHL. Similarly to other studies, eHEALS scores were dichotomized into high EHL (≥26) and low EHL (<26) [13]. Participants received incentives (cookbook, cash card [US $50], heart health book, personal physical activity monitor (Fitbit Charge) at enrollment and were provided with an iPad mini tablet device installed with the app software for use throughout the study. Two one-hour, hands-on instructional training sessions on the app log-in access and the basic app features and navigation were delivered by the study team (one held each in Rochester and MSP, MN). Participants were provided with an instructional manual including step-by-step screen shots to support their independent use.

Inclusion criteria were the following: AA, aged ≥18 years, basic Internet navigation skills, at least weekly Internet access (such as at home, a family member’s or friend’s home, church, library/community center, school/university, Internet café, etc), active email address, minimal fruit/vegetable intake (less than 5 servings/day), no regular physical activity program (less than 30 minutes/day of moderate physical activity), able to engage in moderate physical activity (such as brisk walking, dancing, aerobics, gardening, weight lifting without restrictions including physical disability, use of a wheelchair daily or serious medical condition). Individuals were ineligible if they were unable to walk up at least two flights of stairs or walk at least one city block without assistance or stopping, pregnant, had visual/hearing impairment or mental disability that would preclude independent use of the app or were past participants of the face-to-face CVD prevention program [19]. The three partnering Rochester churches were small in congregation size (varying from 50 to 100 members) and together they constituted approximately 200 congregants (range 50% to 75% adults aged ≥18 years). The two MSP churches were larger in congregation size (varying from 100 to 200 members) constituting approximately 400 congregants (range 50% to 75% adults aged ≥18 years). Thus, as a combination of all five churches, an estimated 300 members comprised our recruitment pool. The Mayo Clinic Institutional Review Board approved the study protocol.

### Community-Based Participatory Research Recruitment Strategy

A series of jointly-led meetings were held over a 6-month period to outline a clear and culturally appropriate plan for recruitment from five local churches. Participating churches designated church liaisons (FAITH! Partners) to engage in these meetings to design and tailor a recruitment strategy. We collaborated with eight FAITH! Partners (seven which were AA women). Each church had at least one FAITH! Partner with three churches designating two representatives. Community kickoff events were suggested by the FAITH! Partners to serve as our primary recruitment tools to outline the study timeline and specific expectations of potential study participants. The FAITH! Partners highly suggested that the study team provide an overview of the mHealth intervention with an emphasis on how the *FAITH! App* was designed and rigorously tested by AA community members to increase its cultural relevance, credibility and uptake. Also, a demonstration of the *FAITH! App* ’s key components was recommended to show their “cutting edge” nature and aim to improve CV health as this was viewed as a way to stimulate excitement among the kickoff event attendees. The study team and FAITH! Partners also felt that connecting CV health promotion to the health assessments was fundamental by highlighting them as a means to “know your numbers” and track them for anticipated improvements over the course of the study. During the kickoff events, it was deemed necessary that the study team clearly describe the requirements of the health assessments and make the events convenient in terms of time and location for our participants (community venues). Furthermore, the health assessments were held at local community health clubs in an aim to increase participant comfortability, awareness, and familiarity with health promotion facilities within their communities.

The FAITH! Partners also recommended that interested participants complete a “Registration/Program Interest Form” at the events. The form included contact information and questions corresponding to inclusion/exclusion criteria. Use of the interest form was affirmed as an essential recruitment tactic by our community partners in order for potential participants to better understand the eligibility requirements and for the study team to gauge the number of eligible participants from each church. The FAITH! Partners also suggested that we utilize self-administered, electronic surveys (rather than written) throughout the pilot study for participant convenience and to facilitate more thoughtful responses. They also felt that participants would find this appealing and would aid in their willingness to enroll in the study.

To remain in alignment with CBPR principles, the group mutually agreed upon stressing the importance of research participation among AAs to advance knowledge on the efficacy of mHealth lifestyle interventions in racial/ethnic minority groups. Efforts were made to underscore how their participation in the study would contribute to the AA community at-large and society as a whole. The presence of the study principal investigator and church leadership (ie, church pastor or FAITH! Partner) at the events was deemed critical by our FAITH! Partners for full transparency and to demonstrate a collaborative and equitable partnership in all research phases—a central CBPR tenet [18,21].

The study team and FAITH! Partners developed uniform recruitment tools including church announcement scripts, flyers and a promotional video containing testimonials from prior study participants and FAITH! Partners regarding the program benefits with integrated motivational spiritual messaging (see Multimedia Appendix 1). Lastly, the group solidified a communication plan to promote the kickoff events, as well as enrollment and attendance at the baseline health assessment through a variety of means of contact (ie, church announcements, flyers, telephone calls and emails). We collectively set a recruitment goal of 50 participants to evaluate the feasibility of our recruitment and intervention approach.

### Pilot Study Recruitment Procedures

FAITH! Partners promoted kickoff events through church announcements and flyers. Three kickoff events were held in June 2016 (two at churches in MSP, MN, one at a community health club in Rochester, MN) and were led by both the study principal investigator (LB) and FAITH! Partners. The events were approximately two hours in duration and held at the convenience of the participating churches (ie, following Sunday worship service or integrated into weekly evening Bible Study). Each event included an introduction to the study team, prior research study findings/accomplishments, an overview of the current research project (timeline, intervention components, health assessments, etc) including the promotional video and open discussion. Healthy refreshments were provided at all events. Interested participants completed the “Registration/Program Interest Form” which was returned to the church designated FAITH! Partner and then forwarded to the study team. Subsequently, the study coordinator contacted the interested participants to reiterate study details and complete eligibility screening. Upon confirmation of eligibility, participants were then invited to the baseline health assessments (two held in July 2016 at community health clubs) and to complete the baseline electronic survey. All participants provided written informed consent at the baseline health assessments.

## Results

**Figure 1:**
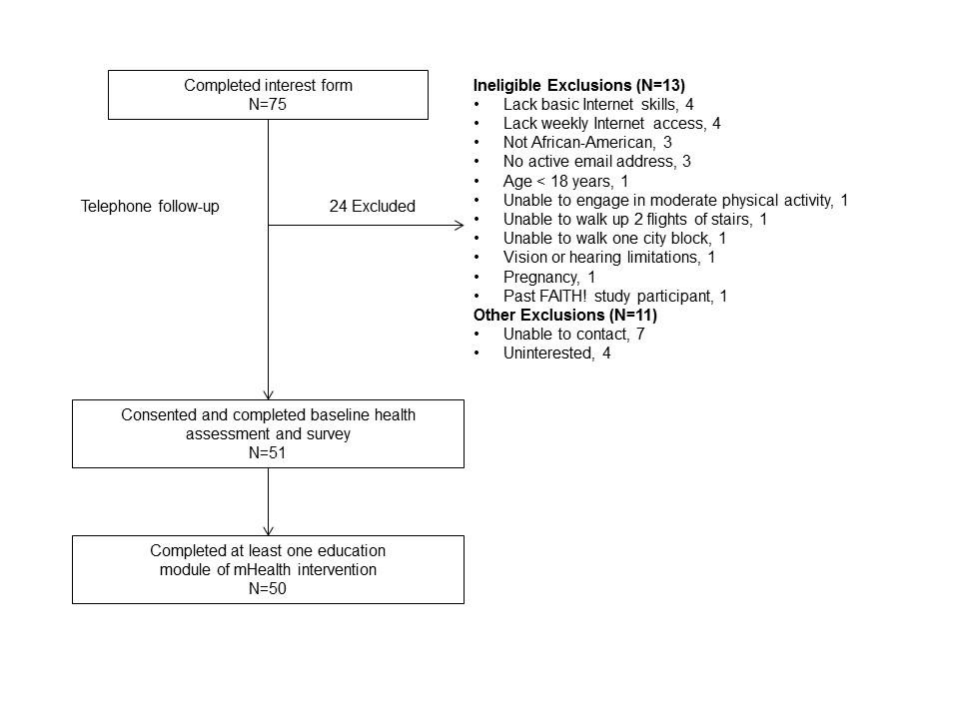
Recruitment process and participant flow. Reasons for exclusion are not mutually-exclusive. One participant withdrew from intervention due to difficulty with the technology.

Approximately 100 individuals attended the three kickoff events. Figure 1 summarizes the recruitment process. Seventy-five individuals completed interest forms and were assessed for eligibility. Of these, 51 (68%) were eligible for participation and enrolled in the baseline health assessment (more than 100% achievement of recruitment goal). Of the 51 enrolled, 100% completed the baseline health assessment and 50 completed at least one education module and form the basis of this report. Of the 24 excluded from the study, 13 (54%) were deemed ineligible by study criteria and 11 were uninterested or unable to be reached by study personnel. The most common reason for ineligibility was lacking basic Internet skills or access (n=4).

Participants were predominately women (70% [35/50]) and employed full time (64% [34/50]) with mean age of 49.6 (SD 12.7) years (Table 1). Nearly all participants used some form of mobile technology (smartphones were the most common, at 92% [46/50]) and reported primary use at home (100%) with typical use in the evening (91.8% [45/49]). The mean EHL score (eHEALS) was 30.4 (range 21-40; SD 4.6), with 84.8% (39/46) having high EHL (score ≥26). There was no difference in mean EHL scores by sex (*P*=0.75). Forty-six out of 50 participants (92%) rated their own ability to use the Internet as ‘fairly skilled to expert’ levels. Only 26% (13/50) reported ‘very often’ downloading apps and 42% (21/50) reported ‘very often’ social media use (data not shown).

**Table 1:** Participant demographics and self-reported mobile technology use.

Characteristic			Total (N=50 unless otherwise noted)
**Sex, n (%)**			
	Female		35 (70.0%)
**Age**			
	Mean (range), years		49.6 (26.0-72.0)
**Marital status, n (%)**			
	Single		9 (18.0%)
	Divorced		7 (14.0%)
	Widowed		2 (4.0%)
	Married/in committed relationship		32 (64.0%)
**Highest level of education, n (%)**			
	Some high school		1 (2.0%)
	High school graduate or GED equivalent		5 (10.0%)
	Some college		12 (24.0%)
	Technical degree or Associate's degree		11 (22.0%)
	College graduate/advanced degree		21 (42.0%)
**Employment status, n (%)**			
	Employed, full-time (32+ hours/week)		34 (68.0%)
	Employed, part-time (less than 32 hours/week)		3 (6.0%)
	Unemployed		17 (34.0%)
**Annual household income (N=49), n (%)**			
	Less than $20,000		5 (10.2%)
	$20,000 to $34,999		9 (18.4%)
	$35,000 to $49,999		10 (20.4%)
	$50,000 to $74,999		9 (18.4%)
	≥$75,000		12 (24.5%)
	Chose not to disclose		4 (8.2%))
**Mobile technology use, n (%)**			
	Smartphones		46 (92.0%)
	Tablet		37 (74.0%)
	Laptop		36 (72.0%)
	Personal physical activity monitor		13 (26.0%)
	No mobile technology use		1 (2.0%)
**Mobile technology use locations (N=49), n (%)**		
	Home		49 (100.0%)
	Family member's home		16 (32.7%)
	Friend’s/neighbor's home		12 (24.5%)
	Work		34 (69.4%)
	Library/community center		6 (12.2%)
	Internet cafe		11 (22.4%)
	School/university		13 (26.5%)
	Church		26 (53.1%)
**Most frequent locations for Internet use (N=49), n (%)**				
	Home		40 (81.6%)
	Family member's home		1 (2.0%)
	Work		7 (14.3%)
	Church		1 (2.0%)
**Average daily mobile technology use (N=49), n (%)**				
	0 to 2 hours		10 (20.4%)
	2 to 4 hours		13 (26.5%)
	4 to 6 hours		8 (16.3%)
	6 to 8 hours		4 (8.2%)
	8 or more hours		14 (28.6%)
**Level of comfort with mobile technology, n (%)**				
	Very comfortable		30 (60.0%)
	Somewhat comfortable		18 (36.0%)
	Neither comfortable nor uncomfortable		2 (4.0%)
**Home Internet access, n (%)**				
	Yes, wireless		47 (94.0%)
	Yes, non-wireless		4 (8.0%)
	No access in home		1 (2.0%)
**Sources primarily used to access health information on the Internet, n (%)**				
	Government websites		15 (30.0%)
	Non-profit organization websites		17 (34.0%)
	Hospital/Clinic websites		31 (62.0%)
	Commercial websites		26 (52.0%)
	Non-Medical websites		11 (22.0%)
	Do not access health information on the Internet		4 (8.0%)
**How useful do you feel the Internet is in helping you in making decisions about your health? (N=46), n (%)**				
	Not useful		1 (2.2%)
	Unsure		7 (15.2%)
	Useful		30 (65.2%)
	Very useful		8 (17.4%)
**How important is it for you to be able to access health resources on the Internet? (N=46), n (%)**				
	Not important		3 (6.5%)
	Unsure		2 (4.3%)
	Important		25 (54.3%)
	Very important		16 (34.8%)
**eHealth literacy score (possible range 8-40, N=46)**				
	Mean (SD)		30.4 (4.6)
	Range		(21-40)
	Low (<26), n (%)		7 (15.2%)
	High (≥26), n (%)		39 (84.8%)
**How would you rate your own ability to use the Internet?, n (%)**				
	Not very skilled		4 (8.0%)
	Fairly skilled		17 (34.0%)
	Very skilled		22 (44.0%)
	Expert		7 (14.0%)

## Discussion

This paper outlines the successful application of a CBPR approach to “re-design” a highly accepted, yet resource-intensive face-to-face program to promote CV health within an mHealth intervention to support broader dissemination capability for the AA community. Leveraging the expertise and insights of our community partners in development of the intervention and the recruitment strategy was crucial to the success of our recruitment efforts. By doing so, we were able to keep the needs of our prioritized population at the forefront which facilitated our participant enrollment. Our investment of resources in face-to-face engagement through kickoff events was important to this community and enhanced transparency and mutual understanding of the intervention goals. Consistent with prior studies of AA adults [2,6], nearly all of our participants reported use of mobile technology (specifically, a high usage of smartphones), a desire to have access to Internet-based health information, and demonstrated high EHL; these factors likely facilitated their enrollment.

A recent systematic review revealed the low representation of AAs in mHealth research [1]. Culturally insensitive recruitment methods and retention strategies have been postulated as potential root causes to low enrollment over apathy from AAs. The review also highlighted the challenge of engaging AA men in these studies and a general increased willingness by AA women to participate in mHealth studies. To overcome this barrier, AA women could serve as advocates to increase AA interest in digital interventions and mHealth research. Our study incorporated the input and active presence of church liaisons (FAITH! Partners) whom were mostly AA women, into designing a culturally relevant intervention and recruitment plan. Their positive influence undoubtedly contributed to us meeting our enrollment goals. The predominance of AA women in our study is reflective of the demographics of the partnering church congregations, and suggests that additional efforts may be needed to reach AA men. It is also noteworthy that the principal investigator is an AA woman and CV medicine specialist; her involvement likely conveyed authenticity, personability and cultural relevance to potential participants. This is in direct congruence with the preferences of racial/ethnic minority groups to include culturally-matched research personnel in leadership roles within clinical studies [26].

To foster recruitment of racial/ethnic minority populations into clinical research whether technology-based or not, it takes an in-depth understanding of their multifactorial, perceived barriers and facilitators to research participation. Having the intent at conception and design of a study to enroll minorities and not having to make midstream adjustments to recruitment efforts have been associated with minority recruitment success rates among study principal investigators [27]. In a recent systematic review of shared barriers and facilitators to research participation among minority groups, the most commonly reported perceived barrier was mistrust in relation to purposeful mistreatment or experimentation by the study investigators [26]. AAs in particular have reported a fear of being treated as “lab rats” or “guinea pigs” which stems from lingering effects related to dark historical unethical and exploitative research practices (eg, US Public Health Services Syphilis Study at Tuskegee, Henrietta Lacks “immortal” cell line) [26,28]. Our study team and community partners wholeheartedly recognized these unfortunate events, but sought to overcome this eroded trust in clinical research and medicine by instilling full transparency and involvement of the community for which our intervention was originally developed into the entire research process. We also made it our goal to underscore the altruistic benefits yielded by the community and future generations from our participants’ research involvement—an articulated facilitator to research participation by minority groups. We devoted a great deal of time explaining the “big picture” of eradicating CV health disparities through our innovative mHealth intervention. We made it clear that there was a growing body of evidence showing the benefits of mHealth interventions in CVD [29], but unfortunately these were not being designed and tested specifically for AAs and others who need them most—underserved racial/ethnic minority populations. From our observation and anecdotally from our community partners and participants, it seemed as if our participants enrolled not only to improve their individual CV health, but also to avoid creating disparities themselves by not participating in a study that was potentially on the frontier of new discoveries within their communities.

When translating community-based, face-to-face health programs to mHealth interventions, it is advantageous to have community “buy in” for trust-building and to overcome challenges to enrollment [1]. The AA church is one of the pillars of the AA community and has a rich legacy central to addressing health disparities among AAs through effective health promotion programming with many engendered through academic-community partnerships [18]. Our ongoing relationship with the AA faith community generated a joint motivation to advance the reach of our CVD prevention program through mobile technology as an mHealth intervention promoting CV health. The collective feedback received from our partnering church leaders and FAITH! Partners assisted in framing a recruitment strategy that was attentive to the facilitators of enrolling AA congregants into our study. The congregations’ established social networks and infrastructure were undoubtedly crucial to our recruitment success as they were the basis for and enabled our community engagement.

### Limitations

We acknowledge limitations to our recruitment methods. We did not collect detailed data on the time and number of attempts required by our study coordinator to reach prospective participants, yet this information would assist other investigators seeking to recruit from AA faith communities by providing them with an estimate of the time allocation required [17]. In the future, we plan to integrate these assessments into our recruitment evaluation strategies. Furthermore, we did not evaluate satisfaction with the recruitment strategy and process from the church pastors or FAITH! Partners immediately following recruitment. These data will be collected in subsequent assessments including focus groups. However, based on their input, a study-specific community steering committee comprised of community and faith-based organizations as well as past study participants has been established to enhance future recruitment strategies. Moreover, we did not include an objective measure of skill assessment to use mobile technologies. However, the fact that our intervention was born out of a community-originated recommendation to develop an mHealth intervention suggests that the group possesses a strong interest in increasing its skillset to use these technologies. In addition, our app was designed through a rigorous formative research process with preliminary usability testing with AA community members of similar demographics to those we intended to recruit into the pilot study. We plan to assess these skills and proficiencies with previously validated tools in future studies.

### Conclusions

Our study supports the effectiveness of integration of a CBPR approach to translate culturally relevant mHealth lifestyle interventions to AAs to maximize recruitment success. Our results also contribute to the growing evidence that AAs have high EHL, are high-users of mobile technologies, and thus are likely to participate in mHealth interventions.

## Appendix

Multimedia Appendix 1 FAITH! program promotional video.

## Abbreviations

AA: African-American
BMI: body mass index
CV: cardiovascular
CVD: cardiovascular disease
CBPR: community-based participatory research
EHL: eHealth literacy
eHEALS: eHealth Literacy Scale
FAITH: Fostering African-American Improvement in Total Health
mHealth: mobile health

## References

[1] James D, Harville C, Sears Cynthia, Efunbumi Orisatalabi, Bondoc Irina (2017). Participation of African Americans in e-Health and m-Health Studies: A Systematic Review. Telemed J E Health.

[2] James DC, Harville C (2016). eHealth Literacy, Online Help-Seeking Behavior, and Willingness to Participate in mHealth Chronic Disease Research Among African Americans, Florida, 2014-2015. Prev Chronic Dis.

[3] Schneider J, Makelarski JA, Van Haitsma M, Lipton RB, Abramsohn E, Lauderdale DS, Lindau Stacy Tessler (2011). Differential access to digital communication technology: association with health and health survey recruitment within an African-American underserviced urban population. J Urban Health.

[4] Watson B, Robinson DH, Harker L, Arriola Kimberly R Jacob (2016). The Inclusion of African-American Study Participants in Web-Based Research Studies: Viewpoint. J Med Internet Res.

[5] Ray R, Sewell A, Gilbert K, Roberts Jennifer D (2017). Missed Opportunity? Leveraging Mobile Technology to Reduce Racial Health Disparities. J Health Polit Policy Law.

[6] Pew Research Center, 2009.

[7] Pekmezi D, Williams D, Dunsiger S, Jennings E, Lewis B, Jakicic J, Marcus Bess H (2010). Feasibility of using computer-tailored and internet-based interventions to promote physical activity in underserved populations. Telemed J E Health.

[8] Joseph R, Pekmezi D, Dutton G, Cherrington A, Kim Y, Allison J, Durant Nefertiti H (2016). Results of a Culturally Adapted Internet-Enhanced Physical Activity Pilot Intervention for Overweight and Obese Young Adult African American Women. J Transcult Nurs.

[9] Yingling L, Brooks A, Wallen G, Peters-Lawrence M, McClurkin M, Cooper-McCann R, Wiley KJ, Mitchell V, Saygbe J, Johnson Twanda D, Curry Rev Kendrick E, Johnson Allan A, Graham Avis P, Graham Lennox A, Powell-Wiley Tiffany M (2016). Community Engagement to Optimize the Use of Web-Based and Wearable Technology in a Cardiovascular Health and Needs Assessment Study: A Mixed Methods Approach. JMIR Mhealth Uhealth.

[10] Abebe N, Capozza K, Des JT, Kulick D, Rein A, Schachter A, Turske Scott A (2013). Considerations for community-based mHealth initiatives: insights from three Beacon Communities. J Med Internet Res.

[11] Buis L, Artinian N, Schwiebert L, Yarandi H, Levy Phillip D (2015). Text Messaging to Improve Hypertension Medication Adherence in African Americans: BPMED Intervention Development and Study Protocol. JMIR Res Protoc.

[12] Joseph RP, Keller C, Adams MA, Ainsworth Barbara E (2015). Print versus a culturally-relevant Facebook and text message delivered intervention to promote physical activity in African American women: a randomized pilot trial. BMC Womens Health.

[13] Norman C, Skinner Harvey A (2006). eHealth Literacy: Essential Skills for Consumer Health in a Networked World. J Med Internet Res.

[14] Mitsutake S, Shibata A, Ishii K, Oka Koichiro (2016). Associations of eHealth Literacy With Health Behavior Among Adult Internet Users. J Med Internet Res.

[15] Richtering S, Hyun K, Neubeck L, Coorey G, Chalmers J, Usherwood T, Peiris D, Chow C, Redfern Julie (2017). eHealth Literacy: Predictors in a Population With Moderate-to-High Cardiovascular Risk. JMIR Hum Factors.

[16] Holt C, Graham-Phillips A, Daniel MC, Slade J, Savoy A, Carter Roxanne (2017). Health Ministry and Activities in African American Faith-Based Organizations: A Qualitative Examination of Facilitators, Barriers, and Use of Technology. J Health Care Poor Underserved.

[17] Hartlieb K, Jacques-Tiura A, Naar-King S, Ellis D, Jen K, Marshall Sharon (2015). Recruitment strategies and the retention of obese urban racial/ethnic minority adolescents in clinical trials: the FIT families project, Michigan, 2010-2014. Prev Chronic Dis.

[18] Coughlin S, Smith Selina A (2017). Community-Based Participatory Research to Promote Healthy Diet and Nutrition and Prevent and Control Obesity Among African-Americans: a Literature Review. J Racial Ethn Health Disparities.

[19] Brewer L, Balls-Berry J, Dean P, Lackore K, Jenkins S, Hayes Sharonne N (2017). Fostering African-American Improvement in Total Health (FAITH!): An Application of the American Heart Association's Life's Simple 7™ among Midwestern African-Americans. J Racial Ethn Health Disparities.

[20] Brewer L, Morrison E, Balls-Berry J, Dean P, Lackore K, Jenkins S, Cohen C, Johnson J, Ellis F, Mangum D C, Hayes Sharonne N, Patten Christi (2017). Preventing cardiovascular disease: Participant perspectives of the FAITH! Program. J Health Psychol.

[21] Israel B, Eng E, Schulz A, Parker E (2005). Methods in community-based participatory research for health, Second Edition.

[22] (2000). National Cancer Institute.

[23] Kim Y, Park I, Kang Minsoo (2013). Convergent validity of the international physical activity questionnaire (IPAQ): meta-analysis. Public Health Nutr.

[24] Norman G, Carlson J, Sallis J, Wagner N, Calfas K, Patrick Kevin (2010). Reliability and validity of brief psychosocial measures related to dietary behaviors. Int J Behav Nutr Phys Act.

[25] Carlson J, Sallis J, Wagner N, Calfas K, Patrick K, Groesz L, Norman Gregory J (2012). Brief physical activity-related psychosocial measures: reliability and construct validity. J Phys Act Health.

[26] George S, Duran N, Norris Keith (2014). A systematic review of barriers and facilitators to minority research participation among African Americans, Latinos, Asian Americans, and Pacific Islanders. Am J Public Health.

[27] Williams I, Corbie-Smith Giselle (2006). Investigator beliefs and reported success in recruiting minority participants. Contemp Clin Trials.

[28] Corbie-Smith G, Thomas S, St George Diane Marie M (2002). Distrust, race, and research. Arch Intern Med.

[29] Kelli H, Witbrodt B, Shah Amit (2017). The future of mobile health applications and devices in cardiovascular health. Euro Med J Innov.

